# Beyond plasmid addiction: the role of toxin–antitoxin systems in the selfish behavior of mobile genetic elements

**DOI:** 10.1128/jb.00232-25

**Published:** 2025-09-19

**Authors:** Bradd Mendoza-Guido, Keilor Rojas-Jimenez

**Affiliations:** 1 Instituto de Investigaciones en Salud (INISA), Universidad de Costa Rica 27915, San José, Costa Rica; 2 Escuela de Biología, Universidad de Costa Rica 27915, San José, Costa Rica; University of Notre Dame, Notre Dame, Indiana, USA

**Keywords:** phage competition, plasmid competition, molecular symbionts, molecular symbiosis, toxin-antitoxin, MGE selfishness, plasmid addiction

## Abstract

Toxin-antitoxin (TA) systems were initially described as “addiction” modules that promote plasmid maintenance through a post-segregational killing (PSK) mechanism. In this process, the cells are forced to retain plasmids to avoid death caused by the longer half-life of the toxin compared to the antitoxin. However, TA systems have since been widely identified across a broad range of mobile genetic elements (MGEs), suggesting that TA systems support the maintenance of these MGEs within bacterial hosts and contribute to the exclusion of competing MGEs such as plasmids and phages. This perspective highlights their broader role beyond plasmid addiction, functioning as key components in safeguarding MGE persistence and enhancing MGE fitness. Therefore, the concept of “plasmid addiction” should be reconsidered as a subset of a more comprehensive phenomenon referred to as “MGE selfishness,” which more accurately captures the widespread distribution and conserved, self-serving functions of TA systems across diverse MGEs. Additionally, TA systems facilitate the establishment of MGEs as “molecular symbionts” within bacterial cells. While initially considered parasitic, the relationships can evolve to provide mutual benefits for both the MGE and the host. From a gene-centered evolutionary perspective, the proposed molecular symbiosis may progress to a point where most of the MGE’s original content is lost, leaving only essential genes that are retained and functionally co-opted by the host. Further studies should investigate the role of TA systems in MGEs beyond plasmids, as well as their evolutionary trajectories toward specialized functions that may influence the adaptation and evolution of key bacterial groups, including pathogens.

## TOXIN-ANTITOXIN (TA) SYSTEMS ARE NOT FOUND EXCLUSIVELY IN PLASMIDS

The term *plasmid addiction* was originally coined to describe the role of TA systems in ensuring plasmid maintenance within bacterial populations (1). These systems promote plasmid stability through a mechanism known as “post-segregational killing” (PSK), in which cells are forced to retain plasmids carrying both the genes coding to produce the toxin and its corresponding antitoxin. Since the toxin typically has a longer half-life than the antitoxin, loss of the plasmid leads to antitoxin degradation and subsequent cell death due to free toxin activity (2). Although this mechanism remains under debate due to limited experimental evidence, a recent study visualized plasmid loss and the resulting PSK at the single-cell level, providing direct confirmation of TA-mediated cell death (3).

The concept of plasmid addiction has thus shaped the understanding of TA systems, particularly in plasmid biology. However, this plasmid-centric perspective may conceal the broader significance of TA systems across other mobile genetic elements (MGEs). TA systems have been extensively characterized across multiple MGE types, including integrative and conjugative elements (ICEs) (4, 5), bacteriophages (prophages) (6), integrons (7), and transposons (8), where they contribute to their own persistence and dissemination. For example, the ICE*Ssu*HN105 element in *Streptococcus suis* harbors a type II and type IV TA systems that act synergistically to promote ICE stabilization and mediate multidrug resistance. Similarly, the SXT element (an ICE conferring resistance to multiple antibiotics) found in *Vibrio cholerae* isolates contains a TA system that helps minimize the emergence of SXT-free cells (4). In strain N16961 of *V. cholerae*, a TA system has been proposed to stabilize a superintegron located on its second chromosome (7).

Although TA systems often function as defense mechanisms against phage infection, some of them also may play a role in prophage stabilization (9). For instance, prophage-encoded TA systems are thought to stabilize prophages in *Escherichia coli* O157:H7 (10), while a type II TA system in the CP4So prophage of *Shewanella oneidensis* is essential for its maintenance (6). Indeed, a recent large-scale genomic study involving over 34,000 bacterial genomes has suggested that TA systems are equally or even more frequently associated with prophage sequences than with plasmids (11).

TA systems are proposed to have originated through multiple evolutionary events. Although eight distinct TA families have been identified, some share structural similarities despite functional differences, suggesting that different evolutionary pathways have converged to play a common TA role (12). In a recent study, Guan et al. (11) reported that 32.6% of all identified TA loci across bacterial genomes are associated with MGEs, comprising a total of 82,018 TA–MGE relationships. While most TA loci are located within chromosomal regions, they may reside in uncharacterized MGEs or chromosomal islets that have lost other MGE-associated genes, retaining only TA systems (12, 13).

In addition, Guan et al. (11) identified specific patterns in the association of TA systems with MGEs: type II systems were primarily linked to plasmids (but also prevalent in other MGEs), type VII to insertion sequence clusters or transposons, type V to genomic islands, and types VI and VIII to prophage sequences. Notably, type VI systems were observed in only three instances across all genomes, with two located within prophages. In Table 1, we show the different known TA systems, describing the nature of their toxins and antitoxins, as well as their associations with various MGEs, according to Guan et al. (11) (see Fig. 2 in their paper).

**TABLE 1:** Overview of the eight types of TA systems, highlighting the nature of their toxins and antitoxins, and their associations with different mobile genetic elements (MGEs)^*a*^

TA type	Toxin	Antitoxin	MGE association	Refs.
I	Small hydrophobic proteins that cause membrane depolarization and ATP loss (with some exceptions)	Small antisense RNAs that interrupt toxin transcription	Mainly with prophages and plasmids, but also with genomic islands, ICEs, and IS/Tn clusters	(14)
II	RNases with varying specificity mainly inhibit peptidoglycan and lipopolysaccharide synthesis, leading to cell wall integrity loss. Some impair DNA replication.	Proteins that bind to and sequester toxins, preventing interaction with their targets	Mainly with plasmids, but also with genomic islands, IS/Tn, IS clusters, ICEs, and integrons	(15–18)
III	Proteins that degrade free mRNA	Pseudoknot RNA antitoxins that bind to and sequester their cognate toxins	Mostly with plasmids (more frequent than in chromosomal regions), and to a lesser extent with ICEs, prophages, IS clusters, and IS/Tn	(19–21).
IV	Proteins that cause DNA damage, inhibit cell division, or induce metabolic stress	Proteins that act on the cellular targets of toxins, protecting or detoxifying them rather than blocking the toxin directly	Mainly with genomic islands, and less frequently with prophages, IS/Tn, and ICEs	(22–24).
V	Proteins that lyse cells by damaging membranes, similar to type I TA systems	Endoribonuclease antitoxins that specifically degrade toxin-encoding transcripts	Mainly with genomic islands, and rarely with IS/Tn	(25)
VI	Proteins that inhibit DNA replication elongation by interacting with DNA polymerase	Protein adaptors that direct toxins for degradation by ATP-dependent proteases	Only three records: two associated with prophages and one with a chromosomal region	(26)
VII	Proteins that Disrupt tRNA function by either ligating pyrimidines to acceptor stems or cleaving the acceptor stem of specific tRNAs	Proteins that inactivate toxins through post-translational modifications (27–29)	Mostly with IS clusters/Tn (more frequent than in chromosomal regions), and less often with plasmids, genomic islands, IS/Tn, and ICEs	(27, 30)
VIII	RNA molecules that sequester rare tRNAs, causing growth arrest	RNAs that repress toxin expression either as antisense RNAs or by mimicking CRISPR RNAs that recruit Cas proteins as transcriptional repressors	Mainly with prophages, and rarely with genomic islands and IS/Tn	(31)

^*a*^ All MGEs associations were adapted from Guan et al. (11) and data available in TADB 3.0 database (https://bioinfo-mml.sjtu.edu.cn/TADB3). Key references for toxin and antitoxin biology across different TA types are indicated in the table.

A type VIII TA system (*creTA*) has been proposed to render CRISPR-Cas systems addictive to their host, thereby promoting the retention of CRISPR-Cas loci in bacterial genomes (31). Since CRISPR loci have also been linked to MGEs known as “casposons” (32), this represents another example of how TA systems contribute to the maintenance and stability of MGEs beyond plasmids, influencing their host fitness. This kind of relationship supports the idea that specific TA system types are preferentially associated with certain classes of MGEs, likely resulting from multiple origination processes. These examples (reviewed by Jurėnas et al. [12]) underscore the broader evolutionary and functional relevance of TA systems beyond plasmid maintenance, also contributing to the stability and success of other MGEs from all types.

## TA SYSTEMS CONTRIBUTE TO MGE SELFISHNESS BEYOND PLASMID MAINTENANCE

The traditional concept of “plasmid addiction” in the context of TA systems suggests that bacteria become dependent on plasmids (or other MGEs) in a manner analogous to drug dependence. Initially viewed as purely selfish genetic elements, TA systems have since been shown to offer benefits to bacteria, potentially contributing to overall population fitness. This dual role was emphasized by Van Melderen and Saavedra De Bast (33), who discussed the widespread maintenance of TA systems in bacterial communities and supporting the perspective that they are more than selfish elements.

Nevertheless, the functional versatility of TA systems in bacteria is not universally conserved. For example, TA systems have been shown to reduce competitive fitness in *Pseudomonas putida* PaW85 (isogenic to KT2440) rather than confer any advantage (34). Moreover, it has been demonstrated that type II TA systems do not induce a persistence state in *E. coli* under antibiotic exposure, as was previously reported (35). These findings support that, although TA systems may offer some advantages (which are discussed later), they largely act as selfish genetic elements, ensuring their own persistence (and that of associated MGEs) through addiction-like mechanisms.

**Fig 1:**
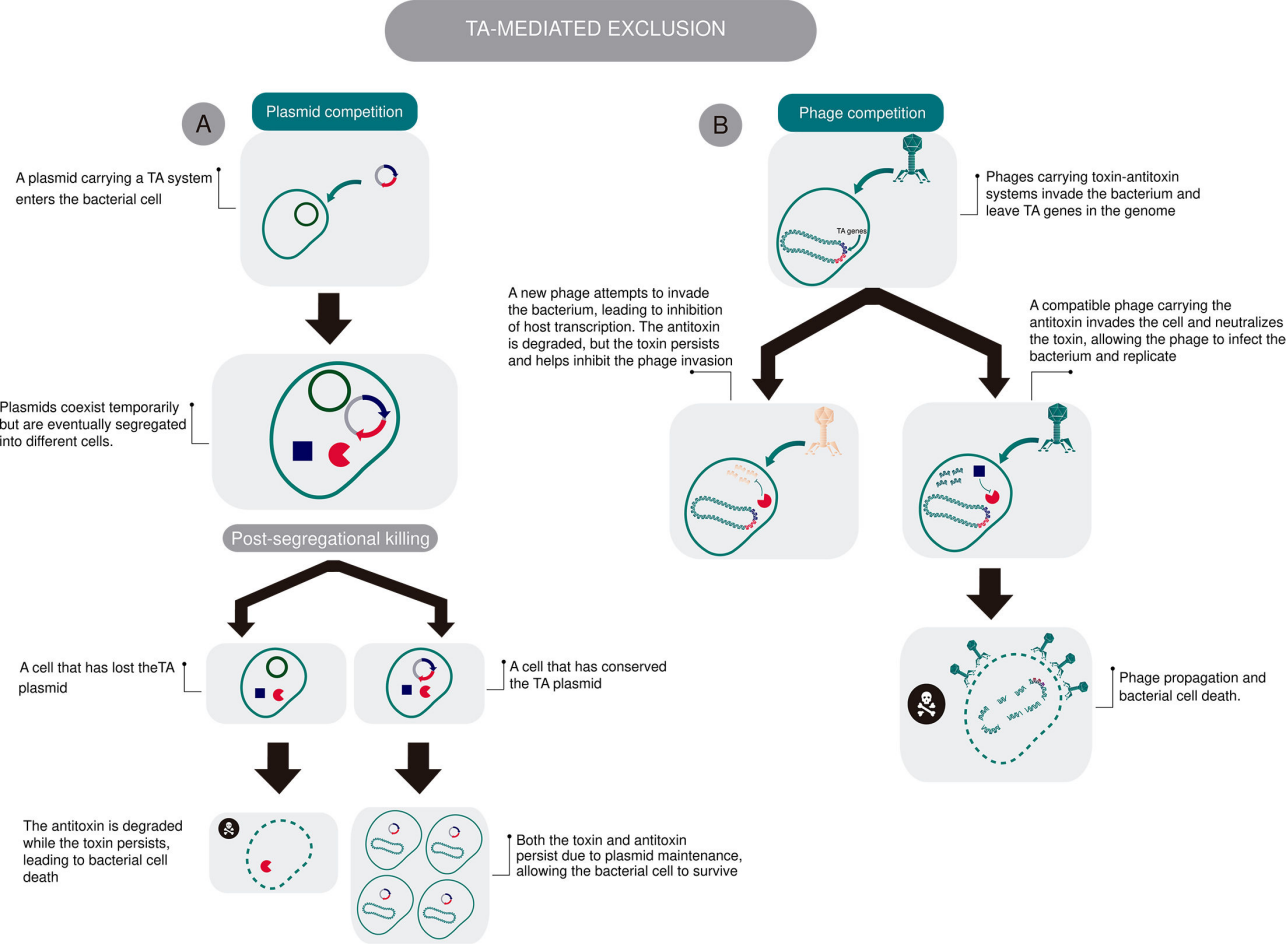
TA-mediated competition in MGEs. (**A**) Plasmid competition model adopted from Cooper et al. (36–38) where post-segregational killing (PSK) of plasmid-free cells arises from plasmid-plasmid competition. Cells that lose the TA-bearing plasmid but retain other plasmids die due to the longer half-life of the toxin compared to the antitoxin, reducing competition and favoring the persistence of TA-positive plasmids. (**B**) Phage competition model proposed in this study, in which phages integrate TA systems into bacterial genomes to ensure the long-term availability of their host by excluding competing phages through toxin-mediated defense. Only phages encoding compatible antitoxins can successfully infect and propagate within the host. Icons were obtained from bioicons.com (39) and edited using Adobe Illustrator 2021.

Notably, TA-mediated selfishness extends beyond plasmid addiction, for example, contributing to the exclusion of other MGEs. Previous studies have shown that TA systems in some plasmids do not necessarily promote plasmid stability within bacterial populations, but instead play a role in excluding plasmids of the same incompatibility group, thereby reducing competition (36–38) (Fig. 1A). Based on these observations, Cooper et al. proposed an alternative model in which TA systems act as a competitive strategy during plasmid co-infection of bacterial hosts. In this model, PSK of plasmid-free cells is not merely a stability mechanism but the outcome of plasmid–plasmid competition: cells that lose the TA-bearing plasmid and retain other plasmids die, thus decreasing competition for TA-positive plasmids (Fig. 1A).

This plasmid-competition hypothesis is supported by one of their studies, in which they observed that TA systems introduced into bacterial populations via transposons were more frequently enriched on plasmids than on chromosomes, suggesting that TA systems are positively selected for their ability to enhance plasmid fitness during within-host competition (38). Interestingly, the fact that some chromosomally encoded TA systems can act as anti-addiction modules, by inhibiting the activity of plasmid-encoded toxins (40, 41), may reflect an exclusion mechanism activated by older MGEs that integrated into the bacterial chromosome but now only the TA system remains (13).

As mentioned, TA systems have been widely recognized as defense mechanisms against phage infections. One such mechanism, known as abortive infection (Abi), relies on the programmed death of the infected host cell by toxin liberation, effectively eliminating the invading phage in an altruistic manner. This mechanism remains debated primarily due to the limited experimental evidence for TA-mediated programmed cell death; however, it is well established that TA systems can confer phage resistance (42–45). Nonetheless, some phages have evolved counter-strategies by encoding their own “antitoxins,” which neutralize host-encoded toxins and enable successful infection (46–48).

For example, the phage ΦTE carries a short sequence highly similar to the RNA antitoxin from a *Pectobacterium atrosepticum* plasmid, which is part of the TA-based phage defense system ToxIN. Mutants of ΦTE phage were observed to acquire a new pseudo-antitoxin sequence, allowing them to evade the host TA system. Notably, these mutants also showed an enhanced ability to transduce replicons expressing the ToxIN TA system, thereby demonstrating phage-mediated horizontal gene transfer and their capacity to mobilize the TA system (and phage defense) to other bacteria (48).

This adaptive mechanism enables certain phages to colonize bacterial cells while the TA system excludes competing phages. The discovery of numerous putative TA systems in prophage regions (11) along with multiple phage-encoded strategies to evade TA systems (49) supports the possibility that these modules represent a strategy by which phages leave behind functional elements (like TA systems) in bacterial genomes (as prophages) to ensure the long-term availability of their bacterial hosts.

Thus, TA systems may play a role in providing defense against a range of phages but may be rendered ineffective against those that carry a similar or compatible antitoxin (Fig. 1B). This hypothesis is supported by the ability of ΦTE to acquire resistance and propagate the ToxIN TA system through transduction; however, it remains speculative and requires further investigation.

The AbiE TA system, which is widespread in bacterial genomes and extrachromosomal elements and has been characterized for its role in phage resistance, also contributes to plasmid stabilization, demonstrating a multifunctional role (42). Another plausible scenario is that such systems originated on plasmids, where they conferred plasmid stability and excluded competitors, while also protecting the host (and thus the plasmid itself) from lytic phages. This trait not only enhances bacterial fitness but also benefits the plasmid, which can persist within the protected host cell. Over time, these mechanisms may have been integrated into bacterial chromosomes, where they serve as widespread antiviral defense systems (13). Nonetheless, as TA systems are thought to have originated through multiple independent events (as mentioned before), that may explain the diverse selfish strategies they have evolved in different MGEs.

In this context, we propose that, rather than viewing bacteria as “addicted” to plasmids or other MGEs, it is more appropriate to consider TA systems as mechanisms that safeguard the persistence of MGEs and help exclude competitors, contributing to MGE fitness. Therefore, the concept of “plasmid addiction” should be reconsidered as a subset of a broader phenomenon referred to as “MGE selfishness” which more accurately reflects the widespread distribution and conserved self-serving functions of TA systems across diverse MGEs.

These TA systems-based strategies allow MGEs to persist within their bacterial hosts and may eventually evolve to confer adaptive traits to those hosts, with MGEs ultimately functioning as molecular symbionts. Nevertheless, further investigation is needed, as most TA systems have been studied primarily in plasmids, while their role and dissemination across other MGEs remain comparatively less studied. Additionally, potential host-imposed constraints that may limit their propagation require further investigation, such as the evolutionary origin of chromosomal TA systems, many of which have been adapted to function as host-mediated anti-addiction mechanisms (28, 40).

## MGEs AS MOLECULAR SYMBIONTS

MGEs have been described as “molecular symbionts” by Filée (29), who specifically highlighted the molecular symbiosis between MGEs and giant viruses (GVs). In this hypothesis, GVs harbor and transmit MGEs in exchange for a defense mechanism provided by restriction-modification (RM) systems encoded by the MGEs. These RM systems function analogously to TA systems: the restriction enzyme protects the virus by cleaving foreign DNA, while the methyltransferase flags the viral genome to prevent self-degradation. The differential stability of the two components, with the restriction enzyme persisting longer than the methyltransferase, creates a dependency of the virus on the MGE, just like the selfish strategy of other MGE carrying TA systems.

Hence, the term *molecular symbionts* (29) can be used to describe how MGEs establish themselves in a form of obligated symbiosis in which bacteria acquire beneficial traits in exchange for maintaining the MGE. However, the introduction and persistence of MGEs is not always voluntary. Maintenance mechanisms such as TA and RM systems can enforce the retention of MGEs through strategies that border on parasitism. In this context, TA modules function as selfish elements that promote the conservation of their associated MGEs while excluding competing elements, effectively taking advantage of their bacterial hosts. Over time, however, these TA systems may evolve to confer additional benefits to the bacterial host, including not only defense mechanisms but also traits such as phage defense, antibiotic resistance, stress tolerance, and, in some cases, evolving as pathogenic effectors (12, 50, 51).

For example, Fic proteins are well-known post-translational effectors employed by many bacteria to modulate host epithelial responses and facilitate colonization (52). However, they have also been identified playing the TA role in *Bartonella* spp. and *Campylobacter fetus* subsp. *veneralis* (51, 53). In *Bartonella*, these Fic proteins are part of TA systems encoded within genomic islands and plasmids that also harbor type IV secretion systems (T4SS). These *Bartonella* TA systems are closely related to the effector protein VbhT, which is secreted by the conjugative Vbh T4SS. Based on this relationship, the authors proposed an evolutionary trajectory in which MGE-encoded TA systems gave rise to interbacterial and inter-kingdom effector proteins, such as the *Bartonella* effector proteins (Beps). These Beps are secreted via the VirB T4SS, another system with an origin linked to MGEs (51).

In this context, it is also possible that a parasitic interaction may eventually evolve into an obligate molecular symbiosis, where bacteria (and viruses) retain MGEs that confer selective advantages. This continuum from parasitism to mutualism (dependent on reaching a stable equilibrium) has been proposed as a framework to explain the emergence of mutualistic relationships across different species, enabling the co-persistence of both partners (54–56). However, many entities continue to maintain parasitic relationships over time, and some are even thought to have originated from mutualistic interactions (a concept known as the mutualism-to-parasitism hypothesis), indicating that the ultimate direction of the relationship is influenced by a variety of factors (57, 58).

**Fig 2:**
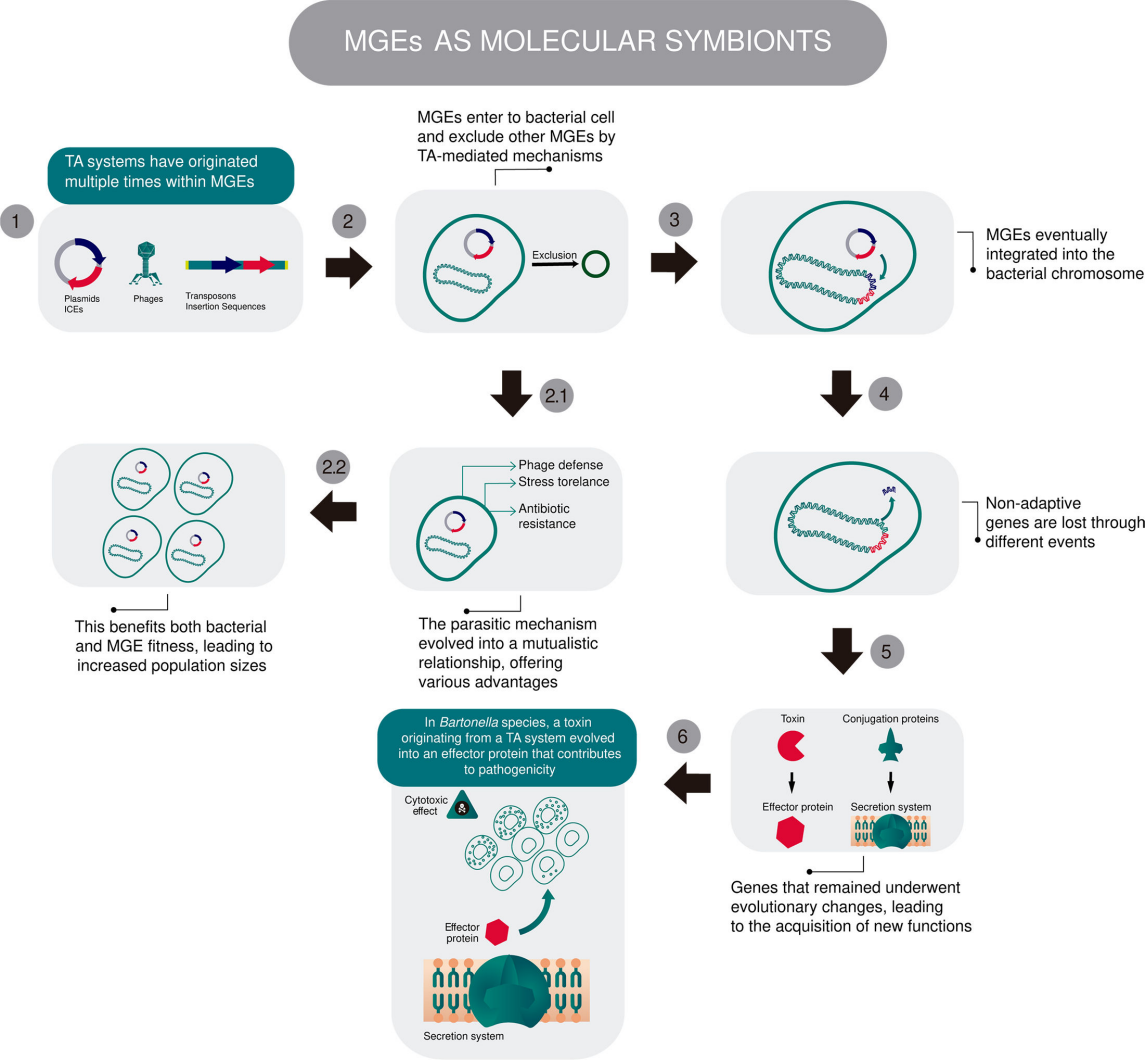
Model proposing the contribution of TA systems to the molecular symbiosis between MGEs and bacteria. TA systems contribute to the exclusion of competing MGEs (as proposed in Fig. 1) through a parasitic mechanism, but in some scenarios, they may also provide distinct advantages to their bacterial hosts, enhancing the fitness of both the MGEs and the bacteria. Subsequently, MGEs may become fixed in the bacterial chromosome (under different conditions), while non-adaptive genes are lost. The remaining genes undergo evolutionary changes and may acquire new functions that benefit bacterial adaptation, for example, the development of effector proteins in *Bartonella* species, which appear to be derived from TA systems. Icons were obtained from bioicons.com (39) and edited using Adobe Illustrator 2021.

Furthermore, some of these relationships between bacteria and MGEs may progress to a terminal stage, in which most of the MGE’s original genetic content is lost. At this point, only a minimal set of essential genes is retained and co-opted by the host, functioning as an independent molecular module (13). This evolutive trajectory resembles the endosymbiont theory (59), which proposes that the ancestral bacteria that gave rise to mitochondria (and chloroplasts) gradually lost much of their genome, retaining only structures and genes essential for their new role within the host cell (Fig. 2).

From a gene-centered view of evolution (60), this outcome represents an evolutionary success: the persistence of specific genes despite the loss of their original MGE suggests the gene’s ability to endure across evolutionary timescales. Indeed, Rocha and Bikard (13) discussed how MGEs can serve as the origin of *defense islands*, integrating into the bacterial chromosome at genomic hotspots. As time progresses, these regions may lose non-adaptive genes through mutation (and other events), while selectively retaining those involved in defense or other beneficial functions. The retained genes can later be repurposed or expanded, evolving toward multifunctional roles, as illustrated by the example of the *Bartonella* effector proteins and their T4SS, which likely originated from MGE-associated TA modules and delivery systems.

## CONCLUSIONS

TA systems, once primarily associated with plasmid maintenance through “plasmid addiction,” are now recognized as widespread components of various MGEs. These systems may act as selfish genetic modules that promote the persistence of their host MGEs through mechanisms such as PSK, MGE exclusion, and defense against competing elements like bacteriophages. Importantly, different TA system types exhibit preferential associations with specific MGE classes, suggesting diverse evolutionary origins and functional adaptations that extend beyond plasmid stabilization.

The shift in perspective, from “plasmid addiction” to “MGE selfishness,” reflects a broader understanding of TA systems as both parasitic and symbiotic molecular entities. Over time, some TA systems may evolve to provide adaptive benefits to their hosts, blurring the line between selfishness and mutualism and reinforcing the concept of MGEs as “molecular symbionts” that contribute to bacterial evolution and ecology. Further studies should investigate the role of TA systems in MGEs beyond plasmids, as well as their evolutionary trajectories toward specialized functions that may influence the adaptation and evolution of key bacterial groups, including pathogens.

Finally, under a gene-centered view of evolution, the integration of MGE-driven TA systems within bacterial genomes could lead to the gradual loss of non-adaptive genes, preserving only advantageous traits that may be repurposed for diverse functional roles. This perspective highlights how selfish genetic elements can fuel bacterial innovation by contributing to genome plasticity and defense potential. Nonetheless, further research is needed to expand the understanding of how TA systems contribute to the selfishness of MGEs beyond plasmids. In particular, experimental studies are crucial to elucidate their precise mechanisms and their impact on bacterial ecology and evolution.
